# Quantifying Perfusion Properties with DCE-MRI Using a Dictionary Matching Approach

**DOI:** 10.1038/s41598-020-66985-9

**Published:** 2020-06-23

**Authors:** Satyam Ghodasara, Yong Chen, Shivani Pahwa, Mark A. Griswold, Nicole Seiberlich, Katherine L. Wright, Vikas Gulani

**Affiliations:** 1 School of Medicine, Case Western Reserve University, Cleveland, Ohio, USA; 2 Department of Radiology, University of North Carolina, Chapel Hill, North Carolina USA; 3 Department of Radiology, Case Western Reserve University, University Hospitals Cleveland Medical Center, Cleveland, Ohio, USA; 4 Department of Biomedical Engineering, Case Western Reserve University, Cleveland, Ohio, USA; 5 Department of Radiology, University of Michigan, Ann Arbor, Michigan USA

**Keywords:** Diagnostic markers, Magnetic resonance imaging, Three-dimensional imaging

## Abstract

Perfusion properties can be estimated from pharmacokinetic models applied to DCE-MRI data using curve fitting algorithms; however, these suffer from drawbacks including the local minimum problem and substantial computational time. Here, a dictionary matching approach is proposed as an alternative. Curve fitting and dictionary matching were applied to simulated data using the dual-input single-compartment model with known perfusion property values and 5 *in vivo* DCE-MRI datasets. In simulation at SNR 60 dB, the dictionary estimate had a mean percent error of 0.4–1.0% for arterial fraction, 0.5–1.4% for distribution volume, and 0.0% for mean transit time. The curve fitting estimate had a mean percent error of 1.1–2.1% for arterial fraction, 0.5–1.3% for distribution volume, and 0.2–1.8% for mean transit time. *In vivo*, dictionary matching and curve fitting showed no statistically significant differences in any of the perfusion property measurements in any of the 10 ROIs between the methods. *In vivo*, the dictionary method performed over 140-fold faster than curve fitting, obtaining whole volume perfusion maps in just over 10 s. This study establishes the feasibility of using a dictionary matching approach as a new and faster way of estimating perfusion properties from pharmacokinetic models in DCE-MRI.

## Introduction

Dynamic contrast-enhanced (DCE) MRI data can be used with a variety of pharmacokinetic models to estimate perfusion properties through either an ROI-based or voxel-based analysis. However, many models are complex, and thus determination of fitted variables can be challenging, particularly when employing voxel-wise analysis as several thousand voxels must be evaluated to estimate many model parameters simultaneously, which poses significant computational burdens^1–5^. Curve fitting algorithms are used to estimate properties of interest^6–8^, but this approach has many potential drawbacks. For instance, these algorithms can be extremely computationally expensive and require many hours to process just one dataset, which has led to the exploration of alternatives such as the linear least squares method^9–11^. Curve fitting algorithms often also have numerous configuration options including initial property guesses, property bounds, algorithm choice, tolerances, and cost functions. The large number of available configuration options and underreporting of precise configurations causes difficulties in replicating perfusion modeling results across institutions. Lastly, curve fitting algorithms are vulnerable to converging on local minima^11^. When this occurs, the best fit to the model is not optimally identified, resulting in an inaccurate estimate of perfusion properties. Furthermore, these occurrences may be difficult to identify.

These drawbacks have significant consequences. Heye, *et al*.^12^ have shown that there is considerable variation in perfusion properties quantified across analysis platforms such as Tissue4D (Siemens, Erlangen, Germany), DynaCAD (Invivo, Gainesville, Florida, USA), Aegis (Sentinelle Medical, Toronto, Ontario, Canada), and CADvue (iCAD, Nashua, New Hampshire, USA). In that study, identical DCE-MRI data were provided to each platform, and all platforms implemented perfusion modeling based on the two-compartment Tofts and Kermode model to characterize uterine fibroids. Several of the final perfusion properties quantified by each perfusion analysis platform showed significant differences, likely due to issues such as required initial guesses, local minima, large number of user-set parameters, and variability in arterial input function (AIF) selection, undermining the effectiveness of perfusion properties as clinical biomarkers and perfusion modeling as a clinical tool.

We demonstrate a dictionary matching approach as an alternative to curve fitting algorithms to estimate tissue properties from complex pharmacokinetic models like the dual-input single-compartment model^6^. This approach is analogous to that used previously in relaxometry and magnetic resonance fingerprinting^13,14^, and it consists of two phases. In the dictionary generation phase, permutations of perfusion properties within specified ranges are applied to a selected pharmacokinetic model, and the outputs are stored in a dictionary. Next, in the dictionary matching phase, acquired contrast agent concentration curves are compared to each entry within the dictionary by using the inner product. The dictionary entry associated with the maximum inner product is considered the best match, and the perfusion properties used to generate that entry will be the best estimates of the true perfusion properties.

Since this dictionary-based approach is relatively new, the first goal of this study is to show that the accuracy of this method is at least equal to that from the more widely-used curve fitting approach^15^. The second goal is to demonstrate the speed advantage of the dictionary method over the curve fitting approach. To achieve these objectives, the two approaches are compared using Monte Carlo simulations and *in vivo* 3D DCE-MRI data from the liver.

## Methods

### Dual-Input Single-Compartment Model

Although a dictionary approach could be used for any DCE application, liver DCE-MRI data were used with a dual-input single-compartment model^6^ (Eq. 1) for this first validation. A dual-input model is necessary to accurately describe tracer kinetics in the liver because it receives blood from two independent vessels: the hepatic artery and the portal vein. The dual-input single-compartment model is described by Eq. 1^6^:

1 $$\begin{array}{c}\frac{d{C}_{L}(t)}{dt}={k}_{1A}{C}_{A}(t-{\tau }_{A})+{k}_{1P}{C}_{P}(t-{\tau }_{P})-{k}_{2}{C}_{L}(t)\end{array}$$

The rate constants (${k}_{1A}$, ${k}_{1P}$, ${k}_{2}$) are commonly reformulated as the arterial fraction (*AF*), distribution volume (*DV*), and mean transit time (*MTT*) as described in Eqs. 2–4^6^:

2 $$\begin{array}{c}AF=\frac{{k}_{1A}}{{k}_{1A}+{k}_{1P}}\end{array}$$

3 $$\begin{array}{c}DV=\frac{{k}_{1A}+{k}_{1P}}{{k}_{2}}\end{array}$$

4 $$\begin{array}{c}MTT=\frac{1}{{k}_{2}}\end{array}$$

*AF* is a dimensionless variable ranging from 0 to 1 and represents the fraction of blood provided by the hepatic artery as opposed to the portal vein. *DV* is the fraction of the liver volume that is accessible to the contrast agent (the gadolinium contrast agent used in this study is unable to be taken up by cells, so this quantity effectively estimates the extracellular space), and it is also a dimensionless variable that ranges from 0 to 1. *MTT* represents the mean time taken for all contrast agent particles to travel from either the hepatic artery or portal venous input through the liver compartment to the hepatic venous outputs, and it is reported in seconds. ${C}_{A}(t)$ and ${C}_{P}(t)$ represent the arterial input function (AIF) and the portal venous input function (PVIF), respectively. Each was generated by placing regions-of-interest in multiple slices of the aorta and portal vein, respectively, and converting signal intensity to concentration. ${C}_{L}(t)$ represents the contrast agent concentration in a unit of liver tissue (e.g. a single voxel or an ROI of many voxels). ${C}_{L}(t)$, ${C}_{A}(t)$, and ${C}_{P}(t)$ are obtained by converting signal intensities obtained from the DCE-MRI experiment into contrast agent concentrations with the AIF and PVIF scaled by $1-\text{hematocrit}$ assuming a hematocrit value of 0.4^1,7^. ${\tau }_{A}$ and ${\tau }_{P}$ are delay properties for the arterial and portal venous input functions, respectively. This study used fixed values for ${\tau }_{A}$ and ${\tau }_{P}$ in accordance with prior studies^8^.

### Curve Fitting

The MATLAB (version 2018b, The Mathworks, Natick, MA) lsqcurvefit function was used to fit the *AF*, *DV*, and *MTT* perfusion properties of the dual-input single-compartment model. This nonlinear least-squares solver uses the trust-region-reflective algorithm with a step tolerance of $1\times {10}^{-2}$, a function tolerance of $1\times {10}^{-3}$, and a minimum gradient change of $1\times {10}^{-1}$. Fitting bounds were set to 0–1, 0–1, and 0.0001–100 with initial property estimates of 0.2, 0.2, and 10 for *AF*, *DV*, and *MTT*, respectively. All other settings were left to their default values (as described in MATLAB’s documentation: https://www.mathworks.com/help/optim/ug/lsqcurvefit.html).

### Dictionary Matching

The first step in creating the dictionary is to select a range of values and a step size for each individual property in the perfusion model to be used. Each permutation of properties is input to the perfusion model to obtain a contrast agent concentration curve, which is then divided by its own L2-norm and stored as one column in the dictionary matrix. The completed dictionary will be size $t\times n$ where $t$ is the number of time frames in the DCE-MRI acquisition and n is the total number of entries in the dictionary.

In the matching phase, signal intensities from the 3D DCE-MRI acquisition are converted to contrast agent concentration curves using computations described previously^7^. Each curve is divided by its own L2-norm and stored as an individual row in a temporary matrix. On completion, this matrix will be $m\times t$ where $m$ is the total number of contrast agent concentration curves. This temporary matrix is then multiplied by the dictionary to obtain a matrix of inner product values with size $m\times n$. The index of the max inner product value is taken for each row to identify the best match dictionary entry, and its associated perfusion properties are considered the best estimate of the true perfusion properties.

In this study, subject-specific dictionaries were created with ranges of 0–1 and 0.0001–100 with step sizes of 0.01 and 1 for *AF* and *MTT*, respectively. *MTT* cannot take on a value of 0 due to Eq. 4, so the lower bound was set to 0.0001. Aside from this, *MTT* only takes integer values. *DV* was fixed to 1 (as it is essentially a scaling property) and later calculated by dividing the L2-norm of the acquired contrast agent concentration (${C}_{L}(t)$) by the L2-norm of the best dictionary match (${C}_{\text{LD}}(t)$). This is described in Eq. 5:

5 $$\begin{array}{c}DV\approx \frac{\sqrt{sum({C}_{L}{(t)}^{2})}}{\sqrt{sum({C}_{LD}{(t)}^{2})}}\end{array}\,$$

A low-rank compressed version of the dictionary was approximated using a randomized singular value decomposition as has been described before in magnetic resonance fingerprinting^16^ due to limitations in computer memory (RAM).

In a separate analysis, the step sizes of *AF* and *MTT* to be used in the dictionary for the primary simulation and *in vivo* analyses were chosen by creating multiple dictionaries with varying step sizes of each perfusion property. Six dictionaries were created with *AF* step sizes of 0.01, 0.02, 0.04, 0.1, 0.2, and 0.5 as well as an additional six dictionaries with *MTT* step sizes of 1, 2, 4, 11, 25, and 50. An idealized contrast agent concentration curve with known perfusion properties was matched to each of these twelve dictionaries in order to assess each step size’s ability to estimate the known perfusion properties. This ensured that reasonable *AF* and *MTT* step sizes were selected in the dictionary method used for the simulation and *in vivo* analyses.

### Monte Carlo Simulations

An idealized contrast agent concentration curve was created from the dual-input single-compartment model using a temporally smoothed AIF and PVIF from a single subject. The curve represented healthy liver tissue with an *AF* of 0.30, *DV* of 0.30, and *MTT* of 30 s^11^. From this single curve, 252 further permutations were created by holding two of the properties constant and varying the remaining property over a range of values. *AF* ranged from 0.2 to 0.7, *DV* from 0.2 to 0.7, and *MTT* from 11 s to 71 s. Each of these curves was then converted to a signal intensity using the spoiled gradient echo equation and white Gaussian noise was added to create 100 noisy signals at each of 10 SNR levels ranging from 10–100 dB in increments of 10 dB.

To generate the noisy curves, the MATLAB function awgn was used. This function starts by measuring the power of the input signal intensity curve using Eq. 6:

6 $$\begin{array}{c}{P}_{Signal}=\frac{1}{t}\mathop{\sum }\limits_{n=1}^{t}signal{(n)}^{2}\end{array}\,$$

With this quantity and the desired SNR, Eq. 7 is used to calculate ${P}_{\text{Noise}}$:

7 $$\begin{array}{c}SNR=10\,\log \,\frac{{P}_{Signal}}{{P}_{Noise}}\end{array}\,$$

Normally distributed random numbers (µ=0, σ=1) were scaled by $\sqrt{{P}_{\text{Noise}}}$ and added to each of the original signal intensity curves to create 100 new noisy signals at each SNR level. Curve fitting and dictionary matching were used to find the best estimate for each noisy signal. The percent error was then calculated between the true perfusion property value and estimates from curve fitting and dictionary matching.

### DCE-MRI Acquisitions

This study is Health Insurance Portability and Accountability Act compliant, and written informed consent was obtained from all subjects. The University Hospitals Cleveland Medical Center Institutional Review Board approved the image acquisition protocol, and all experiments were performed in accordance with the relevant guidelines and regulations.

Perfusion modeling with the dual-input single-compartment model was performed with 3D DCE-MRI data from 3 healthy volunteers and 2 patients with focal liver lesions (metastatic adenocarcinoma and hepatocellular carcinoma) with a Siemens 3 T Skyra scanner. 3D DCE-MRI data were acquired using an accelerated 3D spiral acquisition with gradient and RF spoiling^7^. The acquisition time for each 3D dataset with 60 partitions was 1.6–2.4 seconds, and a total of 100–120 3D volumes were acquired for a scan duration of 3.2–4.0 minutes while the subject breathed freely. After acquiring the first five volumes, one dose (0.1 mmol/kg) of gadobenate dimeglumine (MultiHance; Bracco Diagnostics, Princeton, NJ) was administered at 3 mL/s, followed by 20 mL of saline solution. Additionally, the acquisition used the following imaging parameters: FOV: 38–46 cm; matrix size: 208 × 208 to 240 × 240 (in-plane resolution is effectively 1.9 mm); slice thickness: 3 mm; TR: 4.5–5.1 ms; TE: 0.5 ms; flip angle: 15°; partial Fourier in partition direction: 6/8. On completion of the 4-minute dynamic scan, another 40 second calibration scan was acquired while the subject was freely breathing. This scan included three fully-sampled volumes to extract the GRAPPA weights for image reconstruction^7^.

The raw data from the acquisition were transferred to an offline workstation for post-processing. Spiral GRAPPA was used to reconstruct the undersampled 3D DCE-MRI data^7^. To apply this technique, the calibration scan was used to generate a GRAPPA weight set. These weights were then applied to the undersampled 3D DCE-MRI data to account for the missing spiral interleaves. After GRAPPA reconstruction was completed, images were generated from k-space data by using a non-uniform Fast Fourier Transform (FFT) toolbox for gridding and image reconstruction^17^.

Since these data were acquired while subjects were freely breathing, respiratory motion resulted in inter-frame motion. The data were motion corrected by applying multi-reference image registration using FMRIB’s Non-linear Image Registration Tool (FNIRT)^7,18^. Once motion corrected, the 3D DCE-MRI data were converted to contrast agent concentrations using the spoiled gradient echo signal equation and literature values for blood T_1_ (1800 ms at 3 T), healthy liver T_1_ (800 ms at 3 T), cirrhotic liver T_1_ (950 ms at 3 T), and gadobenate relaxivity (6.3 s^−1^ mM^−1^ at 3 T) ^7,19–21^. A hematocrit of 0.4 was assumed to convert blood concentration to plasma concentration. Perfusion modeling using curve fitting and dictionary matching were then applied on a voxel-by-voxel basis.

To compare perfusion property values between curve fitting and dictionary matching, single-slice regions-of-interest (ROIs) were drawn by a radiologist with 8 years of experience. For healthy subjects, ROIs were drawn in liver parenchyma while subjects with focal liver lesions had ROIs drawn both within the lesions and in liver parenchyma. Since the subject with hepatocellular carcinoma had concurrent cirrhosis, the liver parenchyma ROI represents cirrhotic liver tissue. The mean and standard deviations of each perfusion property were obtained for the voxels within each ROI using curve fitting and dictionary matching. The perfusion properties from each method were further compared using a two-tailed two-sample *t-*test with a p-value less than 0.05 deemed to be significantly different.

### Computation Hardware

All computations were performed on a workstation running Windows 10 with the following specifications: 3.4 GHz Intel Core i5–4670k, 32 GB DDR3 1600 MHz SDRAM (PC3 12800). All code was implemented in MATLAB (2018b).

## Results

**Figure 1:**
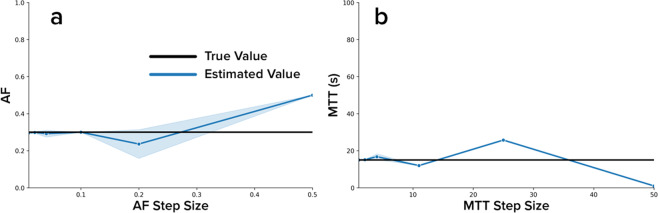
Dictionary Resolution Analysis. Six dictionaries were created with varying step sizes for *AF* as well as an additional six dictionaries with varying step sizes of *MTT*. A simulated contrast enhancement curve with known perfusion properties representing healthy liver tissue was matched against each dictionary. The resulting perfusion property values estimated using the dictionary matching method are plotted against the step size of that perfusion property used to generate the dictionary. The shaded overlays represent one standard deviation of the perfusion property values estimated at each step size. The black horizontal line in subfigure (**a**) represents the true *AF* value of 0.3. The black horizontal line in subfigure (**b**) represents the true *MTT* value of 15 s.

For the dictionary step size analysis, Fig. 1 shows that the dictionary method’s ability to estimate the true perfusion property values diverges as the step size of that property used to generate the dictionary increases. With *AF* and *MTT* step sizes of less than 0.1 and 2 s, respectively, the dictionary method is able to reasonably estimate the true perfusion property values.

**Figure 2:**
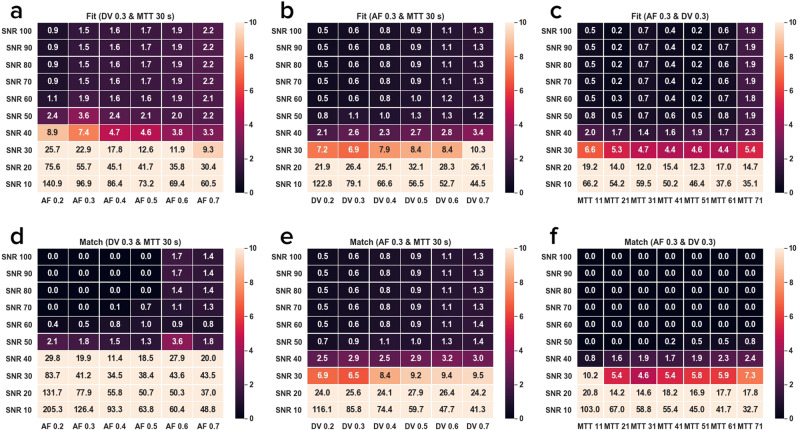
SNR vs. Mean Percent Error of Perfusion Property Estimates from Monte Carlo Simulations. Heatmaps of the mean percent error between the true perfusion property values and estimates using curve fitting (subfigures (**a**), (**b**), and (**c**)) and dictionary matching (subfigures (**d)**, (**e**), and (**f**)) at varying SNR levels. Subfigures (**a**) and (**d**) vary *AF* while holding *DV* and *MTT* constant; subfigures (**b**) and (**e**) vary *DV* while holding *AF* and *MTT* constant; subfigures (**c**) and (**f**) vary *MTT* while holding *AF* and *DV* constant. Each reported mean percent error was taken over 100 noisy curves generated at each SNR.

For the Monte Carlo simulations, Fig. 2 shows heatmaps of the mean percent errors between the correct perfusion property values and estimates from fitting and dictionary matching over each set of 100 noisy curves. Each heatmap shows the mean percent error as both SNR and one of the three perfusion property values are varied using each of the two methods. All six heatmaps show a general reduction in mean percent error as SNR increases. From SNR 60 dB and up, both methods reasonably estimate the *AF*, *DV*, and *MTT*, and there are minimal further reductions in mean percent error for both methods. At SNR 60 dB, the curve fitting estimate had a mean percent error range of 1.1–2.1% for *AF*, 0.5–1.3% for *DV*, and 0.2–1.8% for *MTT*. At the same SNR, the dictionary matching estimate had a mean percent error range of 0.4–1.0% for *AF* and 0.5–1.4% for *DV*. The mean percent error for *MTT* at SNR 60 dB and above was uniformly 0.0% for dictionary matching.

**Figure 3:**
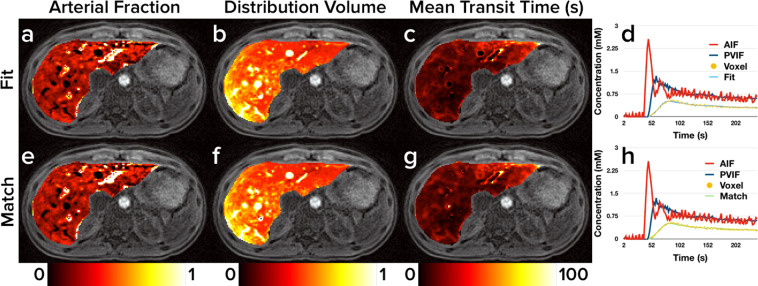
Healthy Volunteer Perfusion Maps. Representative liver perfusion maps from a healthy volunteer (subject 3) with prototypical contrast agent enhancement curves. Maps of each of the three perfusion properties estimated from the dual-input single-compartment model (*AF*, *DV*, and *MTT*) are shown in the first three columns. Perfusion maps from curve fitting are shown in subfigures (**a**), (**b**), and (**c**), and perfusion maps from dictionary matching are shown in subfigures (**e**), (**f**), and (**g**). Subfigures (**d**) and (**h**) show the arterial and portal venous input functions for the volunteer as well as one representative contrast agent enhancement curve. Subfigure (**d**) depicts the estimated curve using curve fitting while subfigure (**h**) depicts the estimated curve using dictionary matching.

**Figure 4:**
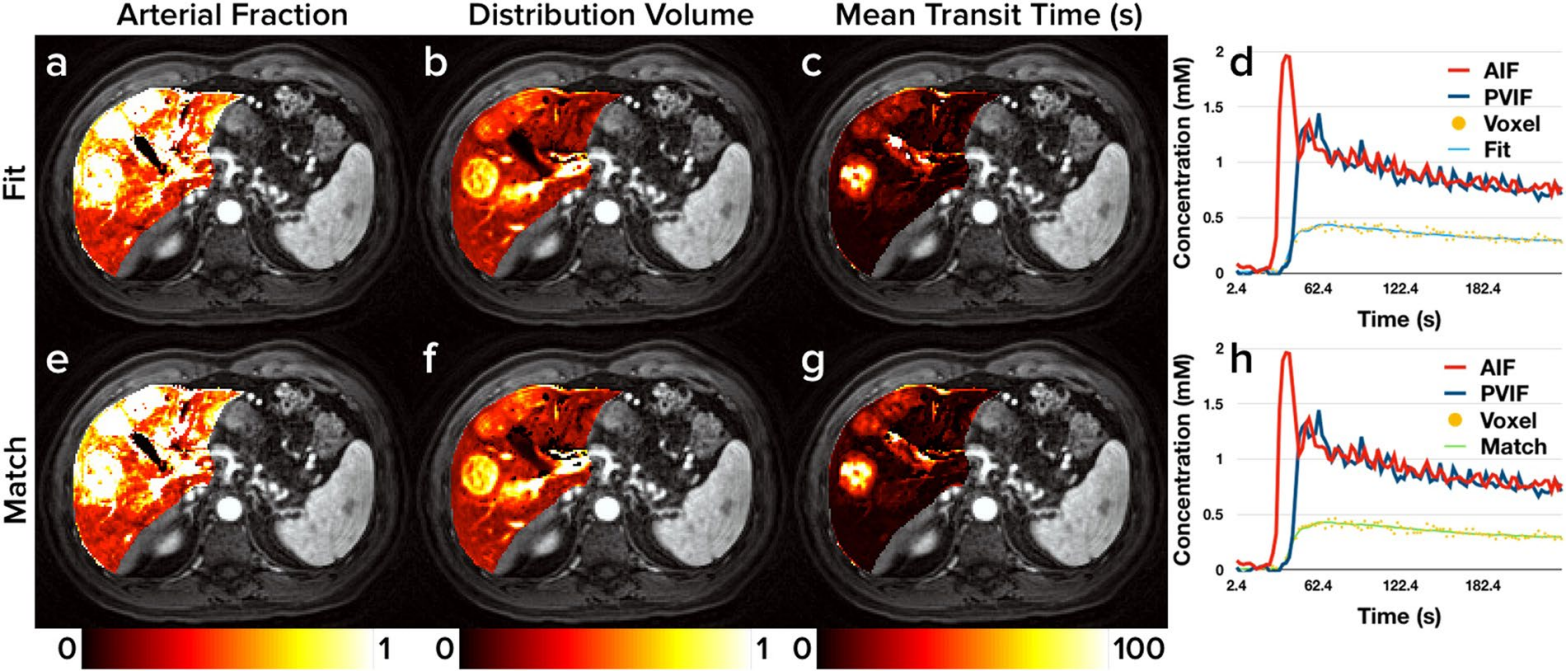
Metastatic Adenocarcinoma Perfusion Maps. Representative liver perfusion maps from a patient with metastatic adenocarcinoma (subject 4) with three lesions visible on the shown slice as well as prototypical contrast agent enhancement curves. Maps of each of the three perfusion properties estimated from the dual-input single-compartment model (*AF*, *DV*, and *MTT*) are shown in the first three columns. Perfusion maps from curve fitting are shown in subfigures (**a**), (**b**), and (**c**). Perfusion maps from dictionary matching are shown in subfigures (**e**), (**f**), and (**g**). Subfigures (**d**) and (**h**) show the arterial and portal venous input functions for the patient as well as one representative contrast agent enhancement curve. Subfigure (**d**) depicts the estimated curve using curve fitting while subfigure (**h**) depicts the estimated curve using dictionary matching.

**Figure 5:**
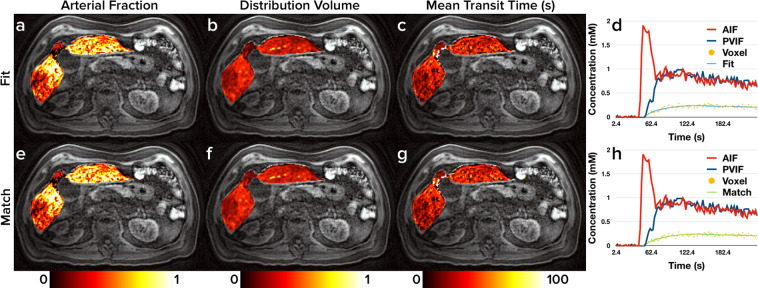
Hepatocellular Carcinoma & Cirrhosis Perfusion Maps. Representative liver perfusion maps from a patient with one HCC lesion and concurrent cirrhosis (subject 5) as well as prototypical contrast agent enhancement curves. Maps of each of the three perfusion properties estimated from the dual-input single-compartment model (*AF*, *DV*, and *MTT*) are shown in the first three columns. Perfusion maps from curve fitting are shown in subfigures (**a**), (**b**), and (**c**), and perfusion maps from dictionary matching are shown in subfigures (**e**), (**f**), and (**g**). Subfigures (**d**) and (**h**) show the arterial and portal venous input functions for the patient as well as one representative contrast agent enhancement curve. Subfigure (**d**) depicts the estimated curve using curve fitting while subfigure (**h**) depicts the estimated curve using dictionary matching.

**Figure 6:**
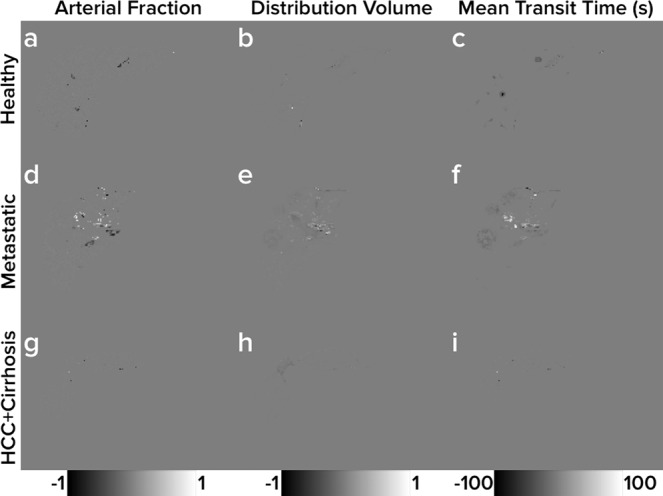
Perfusion Property Difference Maps. Difference maps between the curve fitting and dictionary matching perfusion maps depicted in Figs. 3–5. The three columns show the differences (curve fitting minus dictionary matching) between the three perfusion properties estimated from the dual-input single-compartment model (*AF*, *DV*, and *MTT*). Darker colors indicate dictionary matching has a higher perfusion property value estimate, and lighter colors indicate curve fitting has a higher perfusion property value estimate. Subfigures (**a**), (**b**), and (**c**) in the top row show difference maps from the healthy volunteer from Fig. 3. Subfigures (**d**), (**e**), and (**f**) in the middle row show difference maps from the patient with metastatic adenocarcinoma from Fig. 4. Subfigures (**g**), (**h**), and (**i**) in the bottom row show difference maps from the patient with hepatocellular carcinoma and concurrent cirrhosis from Fig. 5.

Figures 3–5 show the perfusion maps generated from the *in vivo* data using curve fitting and dictionary matching along with representative contrast agent enhancement curves and estimates from each method. Figure 6 depicts difference maps calculated from Figs. 3–5. Table 1 shows a summary of the perfusion properties estimated within ROIs obtained from the *in vivo* data using curve fitting and dictionary matching. Of the 30 measurements made (3 perfusion properties in 10 ROIs), none showed statistically significant differences between curve fitting and dictionary matching.

**Tablee 1:** Perfusion Properties ROI Analysis.

Subject	ROI	Arterial Fraction			Distribution Volume			Mean Transit Time (s)		
		Curve Fitting	Dictionary	p-Value	Curve Fitting	Dictionary	p-Value	Curve Fitting	Dictionary	p-Value
1	Healthy liver	0.28 ± 0.11	0.28 ± 0.11	0.93	0.14 ± 0.06	0.14 ± 0.06	0.91	16.1 ± 3.6	16.1 ± 3.6	0.92
2	Healthy liver	0.23 ± 0.15	0.22 ± 0.13	0.74	0.29 ± 0.04	0.30 ± 0.04	0.75	3.8 ± 1.2	3.7 ± 1.1	0.82
3	Healthy liver	0.19 ± 0.03	0.19 ± 0.03	0.94	0.47 ± 0.03	0.47 ± 0.03	0.85	10.4 ± 0.9	10.4 ± 0.9	0.86
4	Mets lesion 1	0.90 ± 0.17	0.91 ± 0.18	0.54	0.59 ± 0.15	0.61 ± 0.16	0.15	55.3 ± 34.3	58.3 ± 34.4	0.30
	Mets lesion 2	0.96 ± 0.11	0.96 ± 0.11	0.78	0.58 ± 0.12	0.61 ± 0.12	0.21	67.3 ± 25.9	71.4 ± 22.7	0.28
	Mets lesion 3	0.97 ± 0.12	0.97 ± 0.12	0.93	0.64 ± 0.12	0.66 ± 0.12	0.17	51.4 ± 22.8	55.6 ± 21.1	0.09
	Mets lesion 4	1.00 ± 0.00	1.00 ± 0.00	0.31	0.75 ± 0.11	0.77 ± 0.11	0.74	80.4 ± 13.8	80.6 ± 13.6	0.97
	Liver parenchyma	0.34 ± 0.12	0.33 ± 0.13	0.37	0.25 ± 0.08	0.25 ± 0.08	0.59	4.2 ± 1.6	3.9 ± 1.8	0.18
5	HCC lesion	0.92 ± 0.07	0.92 ± 0.07	0.86	0.37 ± 0.09	0.38 ± 0.09	0.81	11.3 ± 7.6	11.5 ± 8.0	0.92
	Cirrhosis	0.33 ± 0.12	0.35 ± 0.13	0.69	0.24 ± 0.02	0.24 ± 0.02	0.47	23.1 ± 4.4	24.4 ± 5.0	0.50

Mean and standard deviation of each of the three perfusion properties (*AF*, *DV*, and *MTT*) estimated voxel-by-voxel within ROIs using curve fitting and the dictionary matching method. The set of perfusion property values from each ROI were compared between the methods, and the p-values of the two-sample *t*-test are reported. The nature of the tissue in each ROI is described under the “ROI” column.

Computational performance between curve fitting and dictionary matching are compared in Table 2. Curve fitting required a mean of 1,500 s (25 min) to generate perfusion maps for whole liver volumes while dictionary matching only required a mean of 10.6 s total (2.4 s for dictionary generation and 8.2 s for matching).

**Table 2:** Perfusion Modeling Speed.

Subject	Curve Fitting (s)	Dictionary		
		Generation (s)	Matching (s)	Total (s)
1	1614	2.7	7.7	10.4
2	1729	2.8	11.6	14.4
3	1347	2.2	6.6	8.8
4	1350	1.9	6.1	8.0
5	1462	2.6	9.1	11.7
Mean	1500	2.4	8.2	10.6

Time required to perform perfusion modeling using the dual-input single-compartment model for whole liver volumes from each of the five subjects studied. Perfusion modeling time for the dictionary method is reported separately for the dictionary generation and matching phases, and their sum is reported in the “Total” column.

## Discussion

The results from Fig. 1 show that as the step size increases for *AF* and *MTT* in the dictionary, the dictionary method’s ability to estimate the true perfusion property values worsens, which is expected. The *AF* estimate is substantially divergent from the true value when using step sizes higher than 0.1, while the *MTT* estimate is substantially divergent when using step sizes higher than 2 s. This study used an *AF* step size of 0.01 and an *MTT* step size of 1 s, which ensured that the subsequent analyses performed were not limited by the resolution of the dictionary.

Curve fitting and dictionary matching are both able to accurately estimate the true perfusion properties in the Monte Carlo simulations from SNR of at least 60 dB. From 60 dB and up, dictionary matching consistently has a slightly lower mean percent error than curve fitting for every tested value of *AF* and *MTT*. For *DV*, the mean percent errors for dictionary matching and curve fitting are nearly identical. Below 60 dB, both methods begin to fail due to higher noise levels. Overall, these results strongly support that both methods are capable of estimating perfusion properties in simulation. With these successful simulation results, the *in vivo* results can be analyzed knowing that any observed differences in this setting are unlikely due to errors in implementation.

Figures 3–5 indicate minimal discrepancies between the perfusion maps generated by curve fitting and dictionary matching, and this is further supported through the difference maps shown in Fig. 6. For the healthy subject depicted in Fig. 3 and subfigures (a), (b), and (c) of Fig. 6, very minor differences are seen largely in the vascular structures where the dual-input single-compartment model does not apply. Similarly, the patient with hepatocellular carcinoma and concurrent cirrhosis depicted in Fig. 5 and subfigures (g), (h), and (i) of Fig. 6 shows almost no differences between the methods. For the patient with metastatic adenocarcinoma depicted in Fig. 4 and subfigures (d), (e), and (f) of Fig. 6, some minor differences are seen around the hilum of the liver as well as the metastatic tumors on the *DV* and *MTT* maps. As the hilum is largely vasculature, the model used in this study would not apply. For the metastatic tumors, the dual-input single-compartment model may also be a poor descriptor of the contrast agent enhancement curve. As these tumors are metastatic, the physiology they exhibit is likely more compatible with their tissues of origin, which often includes colon, breast, or lung. A different physiologic model may be needed to accurately describe these. Thus, the minor differences observed in the perfusion maps are likely a limitation of the model used rather than a limitation of either the curve fitting or dictionary matching approach.

Of the 30 perfusion property measurements made by both the curve fitting and dictionary matching methods in Table 1, zero ROIs showed statistically significant differences, indicating that both methods perform comparably. The mean *AF* measurement of the three healthy liver subjects ranged from 19–28%, which is within the expected range for normal liver tissue^22^. The mean *AF* measurements for all of the metastatic and hepatocellular carcinoma tumors were comparatively very high (over 90% in all cases), which is consistent with the fact that these tumors are known to induce arterial angiogenesis^23^. Interestingly, the liver parenchyma for the patient with metastatic adenocarcinoma (subject 4) exhibits a modestly elevated *AF* compared to healthy liver tissue. This may also be related to arterial angiogenesis. Similarly, in the patient with cirrhosis (subject 5), the cirrhotic liver tissue also exhibited a modestly elevated *AF* compared to healthy liver tissue, which is consistent with the fact that progressive fibrosis of the liver elevates the *AF*^24^.

Comparing time required to perform perfusion modeling for each *in vivo* dataset in Table 2, the dictionary matching approach shows an over 140-fold speedup compared to the traditional curve fitting method. This is particularly beneficial for voxel-based perfusion analysis on 3D DCE-MRI volumes, such as that performed in this study, due to the very large number of contrast agent enhancement curves that must be independently considered. In ROI-based analyses, voxels within an ROI are averaged to generate one contrast enhancement curve that must be estimated. As only one curve must be analyzed, there is limited opportunity for further improvements in speed. Even in pixel-based 2D applications, the number of curves to analyze is substantially smaller than in 3D applications. Similar to ROI-based analyses, the opportunity for further speed improvements is also limited in pixel-based 2D settings. An additional consideration in comparing speed between dictionary matching and curve fitting is the dictionary generation time. More complex pharmacokinetic models with more perfusion properties will require exponentially larger dictionaries. The time saved in using dictionary matching may be offset by the extra time needed to generate the dictionary in this setting.

As the two methods studied in this experiment estimate contrast agent enhancement curves and their associated perfusion properties in fundamentally different ways, they each have their own unique advantages and disadvantages. For example, curve fitting can freely predict any value within specified bounds while dictionary matching is restricted to only providing estimates that exist within the dictionary. Thus, potential values assigned to each pixel are quantized. A drawback of curve fitting is that an initial property guess is required to begin the algorithm, and this guess can influence the final perfusion property estimates^11^. This influence can be profound if there are local minima in the curve being analyzed in this manner. However, there is no reliable method for determining the optimal initial property guess.

One advantage to dictionary matching is the minimal configuration required from the user. Dictionary matching simply requires ranges and step sizes for each property in the model, which is unlike curve fitting and its numerous configuration options. Another advantage is that dictionary matching guarantees an exhaustive search of the dictionary. This means the solution provided by the matching algorithm will be the best possible solution in the dictionary because every dictionary entry is considered as a potential final estimate. As a consequence, dictionary matching is unaffected by the local minimum problem unlike curve fitting, which does not guarantee that every combination of properties within the provided bounds is considered. Despite these benefits, some drawbacks do exist. Primarily, it may be possible for dictionary matching to select a markedly incorrect dictionary entry, but where the signal pattern may superficially be similar to that from the “correct” combination of properties. Although this particular phenomenon was not observed in this study, it is a concern that future users of the dictionary algorithm should note.

Potential users of the dictionary matching algorithm should also note that in its simplest form as demonstrated in this work, the algorithm will guarantee a solution will always be found for each contrast agent enhancement curve matched to the dictionary. Although the properties obtained through matching are the best relative to the rest of the dictionary, they may still provide a suboptimal estimate of the original contrast agent enhancement curve in absolute terms. The inner products obtained from matching contrast agent enhancement curves to the dictionary can be used to quantitatively describe the goodness of fit on an absolute scale from 0 to 1. The inner products could be used to filter out matches that do not meet a specified threshold, or the inner products for a whole dataset could be visualized on a map noting error on a pixel-wise basis.

In this experiment, the dictionary matching approach does appear to provide benefits to speed without sacrificing accuracy in the ROIs analyzed; however, there is substantial room for future work. The dictionary matching technique performs its image analysis after the images themselves have been acquired and transferred to a dedicated workstation for further processing. In many cases, this data transfer time cost is substantial, rendering the improvements in processing time demonstrated in this study insufficient in making liver perfusion MRI a technique that can be applied in a clinically acceptable amount of time. Ideally, whole volume perfusion maps would be generated in near real-time. While the present study is a step in this direction, further development is needed to establish a new workflow that enables routine clinical adoption.

For the simulation analysis performed in this study, the same model was used to generate the idealized contrast agent enhancement curves as was used to assess its performance using both the curve fitting and dictionary matching approaches. This approach will not demonstrate the ability of each method to characterize a more physiologically accurate model, such as those described in^25,26^. Future simulation studies should consider using a more detailed and complex pharmacokinetic model to generate idealized contrast agent enhancement curves. This would provide additional opportunities to examine the benefit of the dictionary-based approach.

Additional improvements can be made in the *in vivo* analysis as well. In this study, each voxel is analyzed independently of its neighbors; however, this does pose a potential problem as individual voxels can be prone to noise. One solution to this is to use spatial regularization techniques to analyze groups of voxels together^27^. This would help boost signal while reducing noise, potentially allowing for a more accurate estimation of perfusion properties by leveraging the information contained in neighboring voxels.

Further studies are also needed to validate the approach across more subjects. Furthermore, alternative algorithms, configurations, and implementations of curve fitting may also provide some boosts to speed and/or accuracy, which should also be compared against the dictionary approach described here. As dictionary matching is analogous to magnetic resonance fingerprinting, there is room to further modify and improve this technique as fingerprinting has through fast group matching^28^ and other developments. With the results of the present study and dictionary matching’s potential benefits, this approach deserves further consideration as an alternative to curve fitting for quantitative analysis of DCE-MRI data.

## Conclusion

This work shows that perfusion modeling can be performed using a dictionary matching approach with markedly shorter computation time and with similar accuracy compared to curve fitting. Dictionary matching circumvents the common problems encountered with curve fitting, namely dependence on initial guesses and potential to select local minima as suboptimal solutions to the fit. However, the algorithm can theoretically select incorrect property estimates if they produce a contrast agent enhancement curve that appears superficially similar to the curve produced by the correct properties, although this was not explicitly observed in the current study. Despite this, the dictionary matching approach may be preferred over curve fitting in certain settings (and vice versa) as the two algorithms pose different tradeoffs. While a dual-input single-compartment model for liver imaging was chosen for demonstration of the concept, such an approach could be applied to DCE-MRI experiments with other perfusion models. The dictionary matching method is an additional promising alternative to curve fitting and warrants further study.

## Data Availability

The simulated and *in vivo* data used in this study are available from the authors on reasonable request.
